# Dietary factors and their influence on immunotherapy strategies in oncology: a comprehensive review

**DOI:** 10.1038/s41419-024-06641-6

**Published:** 2024-04-09

**Authors:** Aleksandra Golonko, Tomasz Pienkowski, Renata Swislocka, Sylwia Orzechowska, Krystian Marszalek, Lukasz Szczerbinski, Artur Hugo Swiergiel, Wlodzimierz Lewandowski

**Affiliations:** 1 Prof. Waclaw Dabrowski Institute of Agricultural and Food Biotechnology State Research Institute, Rakowiecka 36, 02-532 Warsaw, Poland; 2 Clinical Research Center, Medical University of Bialystok, M. Skłodowskiej-Curie 24a, 15-276 Bialystok, Poland; 3 Department of Chemistry, Biology and Biotechnology, Bialystok University of Technology, Wiejska 45 E, 15-351 Bialystok, Poland; 4 Faculty of Chemistry, Jagiellonian University, Gronostajowa 2, 30-387 Krakow, Poland; 5 Faculty of Biology, Department of Animal and Human Physiology, University of Gdansk, W. Stwosza 59, 80-308 Gdansk, Poland

**Keywords:** Tumour immunology, Immunotherapy

## Abstract

Immunotherapy is emerging as a promising avenue in oncology, gaining increasing importance and offering substantial advantages when compared to chemotherapy or radiotherapy. However, in the context of immunotherapy, there is the potential for the immune system to either support or hinder the administered treatment. This review encompasses recent and pivotal studies that assess the influence of dietary elements, including vitamins, fatty acids, nutrients, small dietary molecules, dietary patterns, and caloric restriction, on the ability to modulate immune responses. Furthermore, the article underscores how these dietary factors have the potential to modify and enhance the effectiveness of anticancer immunotherapy. It emphasizes the necessity for additional research to comprehend the underlying mechanisms for optimizing the efficacy of anticancer therapy and defining dietary strategies that may reduce cancer-related morbidity and mortality. Persistent investigation in this field holds significant promise for improving cancer treatment outcomes and maximizing the benefits of immunotherapy.

## Facts

- Specific dietary components (vitamins, fatty acids, and nutrients) can enhance anticancer immunotherapy by modulating immune responses.
- Caloric restriction and fasting-mimicking diets may reduce cancer risk and enhance immunotherapies by promoting immunogenic cell death and sensitizing tumor cells.
- Polyphenolic compounds (resveratrol, apigenin, curcumin) modulate PD-L1 expression, suppressing immune responses against cancer cells and potentially enhancing immune checkpoint therapies.
- Low-protein diets (LPDs) variably affect different cancers, with evidence suggesting they can significantly reduce tumor growth by modulating immune responses and amino acid metabolism.

## Open Questions

- How can future research effectively combine nutritional strategies with immunotherapies to improve their efficacy and reduce the side effects of cancer treatment?
- What approaches can be developed to personalize immunotherapies and dietary interventions based on individual genetic, immunological and metabolic profiles to achieve optimal cancer treatment outcomes?
- How can an improved understanding of the cancer immunity cycle, including dietary influences, lead to a more comprehensive and effective combination?

## Introduction

**Fig. 1:**
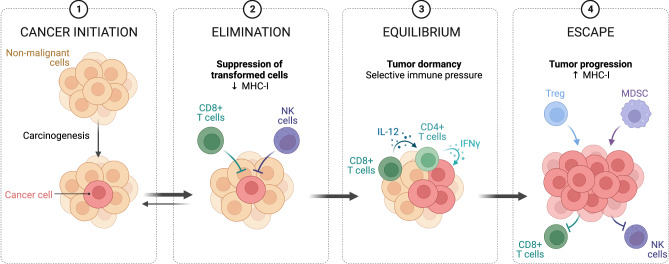
Immune surveillance in controlling malignant cells. This critical process involves the recognition and elimination of various types of cancer cells by different immune system cells. In cases where the elimination process is not adequately effective, mutated cells can evade immune detection mechanisms, leading to uncontrolled proliferation and the formation of a tumor. Created with BioRender.com.

Cancer-related mortality poses a substantial global health challenge, claiming numerous lives annually. The primary culprit behind the fatal outcome for cancer patients is the occurrence of metastases. Metastases result from the migration of primary tumor cells through surrounding tissues and the basal lamina, eluding detection by the host’s immune system and establishing in distant organs. The progression of tumor growth unfolds in three distinct stages (Fig. 1).

The initial phase, termed the “elimination phase,” involves immune cells identifying and eradicating potential cancer cells. If this phase proves ineffective, the tumor advances to the “equilibrium phase.” In this stage, cancer cells gain resistance to detection and destruction, often due to mutations or noncanonical post-translational modifications of membrane proteins. This phase, also known as cancer immunoediting, encompasses the continued proliferation of surviving cancer cells. Ultimately, the tumor enters the “escape phase,” marked by the establishment of an immunosuppressive tumor microenvironment. During this phase, accumulated cancer cells successfully elude immune system responses, culminating in metastases [1]. Swift intervention significantly enhances the likelihood of effective therapy. However, conventional approaches heavily rely on radio and chemotherapy, which can adversely affect the overall health of patients.

Fortunately, the increasing use of immunotherapies provides the prospect of a less toxic therapeutic approach. Recently, there is growing interest in exploring the role of diet in enhancing the immune system, potentially augmenting the efficacy of immunotherapy. Several studies have underscored the connection between dietary patterns and the induction of inflammation, emphasizing the potential impact of dietary modifications on both cancer metabolism and the corresponding immune response [2–5].

The Western diet, characterized by excessive consumption of processed and refined foods, has been linked to elevated inflammation levels. In contrast, diets rich in fruits, vegetables, whole grains, and healthy fats, exemplified by the Mediterranean diet [6], consistently exhibit associations with reduced inflammatory markers. This reduction is attributed to the emphasis on plant-based foods, fish, and olive oil.

In this literature review, our objective is to delineate the impact of specific dietary components that concurrently exhibit anticancer properties. These components hold the potential to offer benefits in cancer prevention while positively influencing anticancer immunotherapy by fortifying the immune system.

## Discussion

**Fig. 2:**
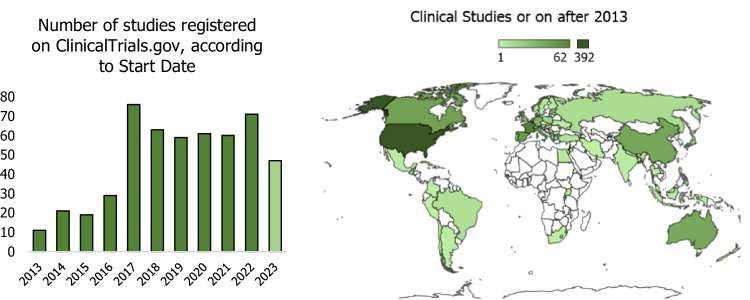
Trends in clinical trials (2013–2023) on nutrition, immunotherapy, and cancer. This chart depicts the number of clinical trials reported by ClinicalTrials.gov, focusing on three keyword groups: (supplementation OR vitamins OR supplement OR nutrition OR diet OR microelements OR food OR macronutrients OR polyphenols OR flavonoids) AND (immunotherapy OR immune system) AND (Cancer OR neoplasm OR tumor), with start dates on or after 01/01/2013.

Over the past decade (2013–2023), there has been a noticeable increase in clinical research integrating immunotherapy, diet, and supplementation in cancer therapy **(**Fig. 2); despite the outbreak of the COVID-19 pandemic in 2019, the number of these clinical trials remained at a constant level, demonstrating resilience and unwavering scientific interest in these research areas even in the face of global health challenges.

### Exploring the landscape of immunotherapies

**Fig. 3:**
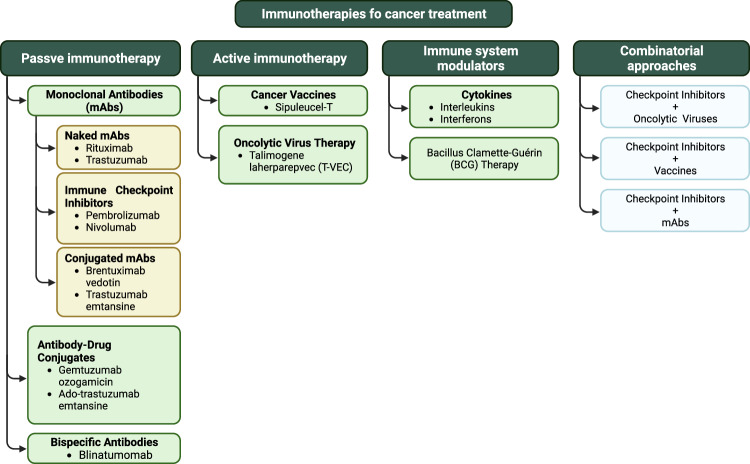
Classification of cancer immunotherapy strategies. Created with BioRender.com.

The cancer immunotherapy landscape is driven by innovative strategies (Fig. 3), including therapeutic monoclonal antibodies with significant antitumor activity. These antibodies stimulate the patient’s immune response. Immune checkpoint inhibitors (ICIs) unshackle T cells, cancer vaccines elicit immune responses against tumor antigens, and adoptive cell transfer techniques utilize T cells to attack tumors. These approaches range from overall immune system activation to precise, targeted actions, some customizable through genetic engineering [7]. Cancer immunotherapy is categorized into passive or active, immune system modulators, and combinatorial approaches. Passive immunotherapy employs monoclonal antibodies or immune cells, while active immunotherapy stimulates the patient’s immune system. Immune system modulators enhance or suppress the immune response, and combinatorial approaches combine different strategies to improve outcomes.

**Fig. 4:**
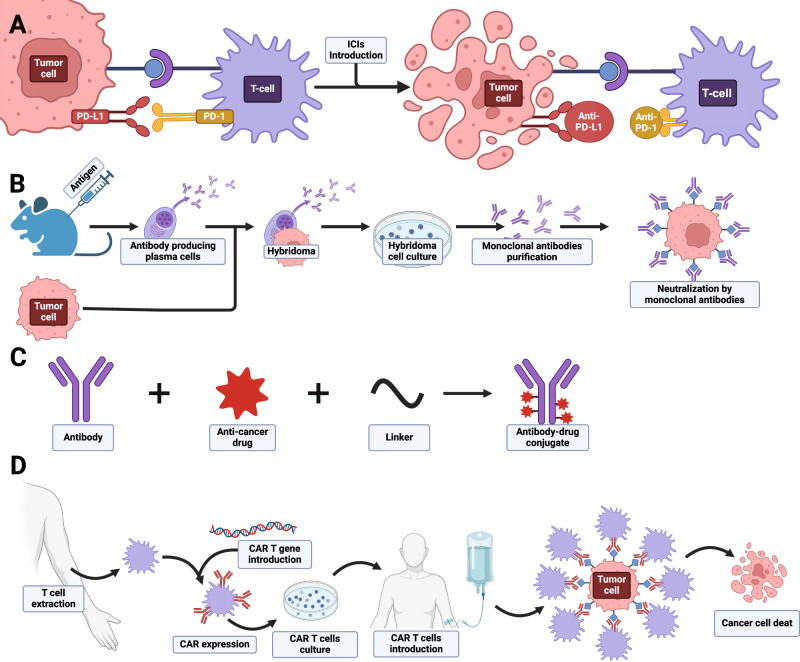
Basic overview of immunotherapeutic approaches utilized in cancer treatment. **A** Immune Chcekpotin Inhibitors; **B** Monoclonal antibodies; **C** Antibody-drug conjugates; **D** Adoptive Cell Therapy.

Monoclonal antibodies (mAbs) have evolved as promising therapeutic agents, designed for specific interactions with cancer cells (Fig. 4B). In clinical practice, examples like trastuzumab (Herceptin) and rituximab (Rituxan) target specific antigens, demonstrating clinical efficacy in breast cancer and lymphoma [8].

ICIs, a significant subgroup of monoclonal antibodies, target immune checkpoint proteins that regulate T-cell activation (Fig. 4A). Commonly employed in cancer treatment, they focus on blocking essential immune checkpoint pathways, restoring proper T-cell function and improving the immune response against cancer cells [9]. Notable ICIs include ipilimumab, nivolumab, pembrolizumab, atezolizumab, durvalumab, and avelumab, targeting pathways such as CTLA-4 and PD-1/PD-L1 [10–15]. Resistance to ICIs can be intrinsic, related to tumor cell alterations, or extrinsic, influenced by factors like the tumor microenvironment, host characteristics, and diet [16].

Antibody–drug conjugates (ADCs) combine monoclonal antibodies with cytotoxic drugs, delivering targeted precision and reducing systemic toxicity (Fig. 4C). ADCs like ado-trastuzumab emtansine and brentuximab vedotin show promise in cancer therapy, targeting specific antigens [17].

Immune cytokines, including IL-2, interferon-alpha (IFN-α), and granulocyte-macrophage colony-stimulating factor (GM-CSF), modulate the tumor microenvironment and activate immune cells. Despite demonstrated clinical benefits, their limited tolerability and severe toxicity hinder standalone use. Research explores combining cytokines with other immunotherapies to address these limitations [18–20].

Adoptive Cell Therapy (ACT) involves extracting, modifying, and reintroducing immune cells to enhance the body’s natural immune response to cancer (Fig. 4D). TCR-engineered T-cell therapy and Tumor-Infiltrating Lymphocytes (TILs) are subsets of ACT, showing promise in treating various cancers. Chimeric Antigen Receptor (CAR) T-cell therapy genetically engineers T cells to recognize specific tumor antigens, demonstrating significant success in hematological malignancies [21–23]. Ongoing research aims to extend ACT benefits to solid tumors, with a focus on personalized medicine and gene editing techniques like CRISPR/Cas9 [24–29].

### The role of diet in immune cell functioning

Obesity and metabolic disorders disrupt the activation and coordination of macrophages, resulting in the loss of their positive influences and the onset of harmful inflammation. Macrophage responses are regulated by two activation programs: classical (M1) and alternative (M2). Classical activation, driven by bacterial infection products, leads to the formation of highly inflammatory macrophages. Alternative activation, triggered by parasitic infections and interleukins, supports antiparasitic functions and tissue repair. The therapeutic implications suggest that targeting macrophage activation, rather than inflammation, could be an effective tool in combating various diseases [30].

Poledne et al. [31] proposed that macrophage polarization in human visceral adipose tissue, linked to fatty (FA) acid metabolism, cell membrane composition, and diet may play a role in inflammation and the risk of cardiovascular disease. FA synthesis in macrophages, promoting M1 polarization in tissue culture, influences macrophage polarization by incorporating FAs into plasma membrane phospholipids. Consequently,elevated palmitate, a product of FA synthesis, may increase the proportion of proinflammatory adipose tissue macrophages in living kidney donors [31].

Chronic inflammation, impacting cancer development, can be influenced by diet. This type of inflammation may not cause noticeable symptoms in the way acute inflammation does, but it can contribute to various chronic conditions and diseases, like cancer Studies indicate that a vegetables-rich diet is linked to a reduced inflammatory profile. The impact of vegetable consumption on lymphocyte counts is mediated by the bacterial genus Collinsella, which tends to increase with the intake of processed foods [32]. In advanced-stage cancer, proinflammatory cytokines contribute to treatment resistance and the development of anorexia, cachexia, and pain [33].

The dietary composition of FAs influences cytokine production. Saturated FAs minimally impact cytokine production, whereas polyunsaturated FAs hinder the production of Th1-type cytokines, known for eliciting proinflammatory responses [34]. Maintaining a balance between Th1 and Th2 responses is crucial for cancers like hepatocellular carcinoma [35].

To establish a distinct classification of foods negatively affecting the body’s inflammatory processes, the Dietary Inflammatory Index (DII) was created. Introduced in 2013, the DII was formulated with the goal of developing a literature-derived, population-based index to assess the inflammatory potential of diverse diets [36]. Incorporating foods with lower DII scores, such as fruits, vegetables, whole grains, lean proteins, healthy fats, and specific spices, can effectively reduce systemic inflammation and mitigate the risk of cancer development [37]. Interestingly, patients with a high body mass index (BMI) were found to have better responses to anti-PD-1/PD-L1 therapy than those with lower BMI [38]. Additionally, diets rich in omega-3 FAs and high-fiber content have been linked to improved outcomes in cancer patients undergoing immunotherapy [39].

These reports highlight a significant link between nutrition and the effectiveness of immuno-oncological treatment. Consequently, alongside observational and interventional studies, there is intensive research and experimention on diagnostic and therapeutic tools. Recent advancements, including single-cell sequencing [40], high-dimensional cytometry [41], and CRISPR‒Cas9 gene editing [42], have revolutionized immunology research, allowing in-depth exploration of immune cell heterogeneity and function. Also, Raman imaging, a label-free and noninvasive technique combined with chemometrics methods, is a potent approach for analyzing dietary-induced biochemical and morphological changes in immune cells (e.g., macrophages, lymphocytes). The advantages of Raman spectroscopy, such as its effective use in cancer cell profiling for routine clinical practice and live-cell imaging, coupled with high sensitivity, chemical specificity, small sample volume, and the ability to measure in liquids with excellent spatial resolution, showcase the substantial potential of Raman spectroscopy for both cancer diagnostics and monitoring immune cell changes [43].

### Dietary and nutritional strategies to enhance immunotherapy effectiveness

**Fig. 5:**
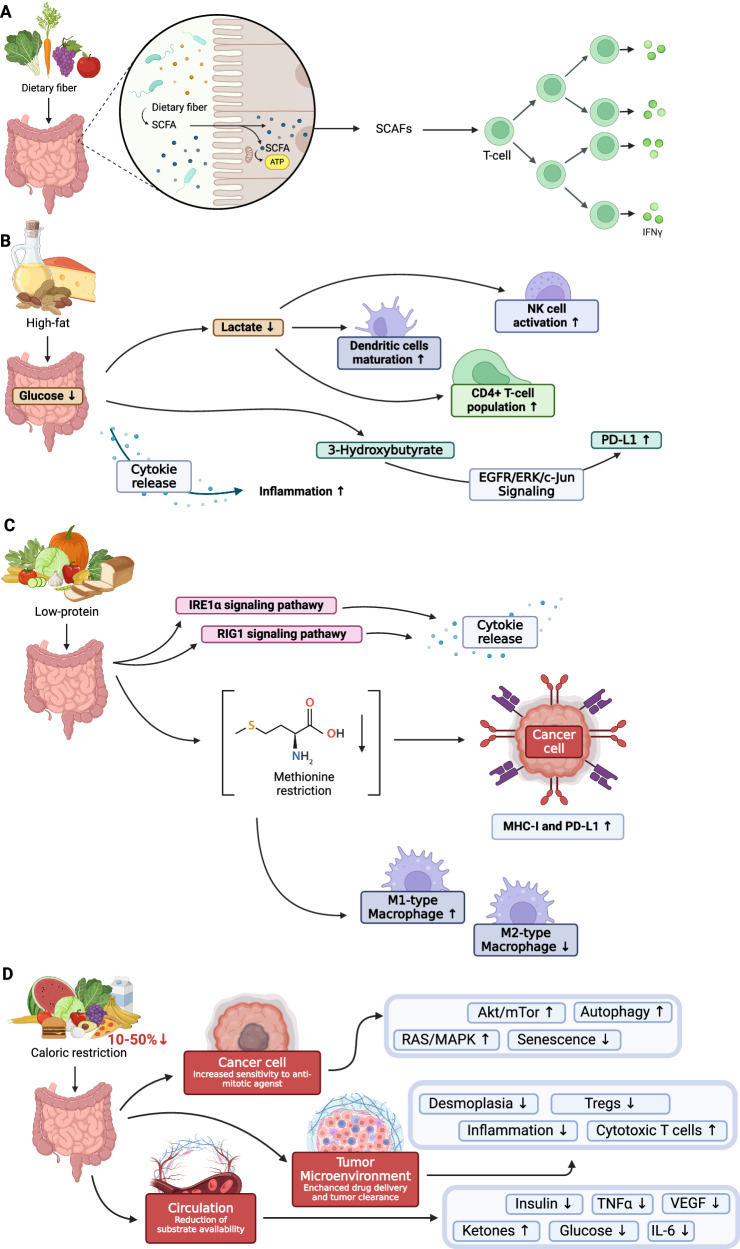
Overview of diet impact on immune system. **A** Impact of diet rich in dietary fiber; **B** Impact of high-fat diet; **C** Impact of low-protein diet; **D** Impact of caloric restriction.

Consuming fibrous foods as prebiotics in the gut influences mucosal immune functions, reducing the risk of enteric inflammation by elevating anti-inflammatory cytokines and decreasing proinflammatory cytokines and the systemic immune response [44]. Undigested food can be converted into short-chain FAs (SCFA), like butyrate, which the gut absorbs, alleviating inflammatory disorders by boosting T-regulatory cell (Treg) numbers and reducing IFN-γ levels. A fiber-rich diet proves beneficial when using ICIs, likely due to the increased SCFAs that stimulate immune cell differentiation and function **(**Fig. 5A**)** [45, 46]. The beneficial effects of dietary fiber consumption stem from its physical, immunomodulatory, and prebiotic properties. Nonfermentable fiber in the diet increases fecal bulk, effectively diluting carcinogenic substances in the gastrointestinal tract. Additionally, fermented dietary fiber reduces fecal pH, diminishing the production of carcinogenic compounds from bile acid metabolism by intestinal bacteria. The immunomodulatory and prebiotic activities of dietary fiber further contribute to its overall positive impact on gut health, underscoring the importance of incorporating fiber-rich foods into a balanced diet [47].

The ketogenic diet (KD) involves obtaining the majority of energy from fat (~75–90% fat) rather than carbohydrates (CHO),in contrast to the typical Western diet (55% CHO, 30% protein, 30% fat) [48]. According to the concept promoting KD in cancer therapy and prevention, reducing carbohydrate intake deplets ATP in cancer cells, while normal cells utilize ketone bodies as an energy substrate. A KD has been observed to lower glucose levels, thereby reducing lactate production by glycolytic cancer cells (Fig. 5B).

Recent studies have broadened the understanding of lactate role in tumor environment, impacting immunological function [49, 50]. Lactate serves as an inhibitor of dendritic cell maturation, hinders NK cell function, thereby limiting innate immunity effectors, and acts as a regulator of gene expression, including NCR1, which encodes a receptor activating NK cells [51]. KD has been found to increase the CD4 + T-cell population and decrease Tregs in animals compared to controls. Additionally, Tregs from KD-fed animals produced less IL-10 when stimulated with tumor cells [52]. KD has emerged as a potential adjuvant in cancer immunotherapy, positively impacting ICI responsiveness. By modulating the expression of immune checkpoint molecules like CTLA-4 and PD-1 on tumor-infiltrating lymphocytes and PD-L1 on tumor cells, KD may enhance ICI-based therapy effectiveness [52] Ferrere et al. [53] study, on mice exhibiting enhanced responses to anti-PD-1 therapy when fed a KD, suggest that it may amplify the anticancer effects of PD-1 blockade, underscoring the impact of diet on the effectiveness of immunotherapy. The antitumor effects of a KD was reversed by T-cell depletion experiments conducted in vivo in mice, suggesting that the diet’s metabolic impact enhances antitumor immunosurveillance, partly through the regulation of immune checkpoint protein expression.

3-Hydroxybutyrate, the principal ketone body, has been shown to induce T-cell-dependent tumor growth retardation in aggressive tumor models, even when standard therapies fail (Fig. 5B**)**. This antineoplastic effect prevents immune checkpoint blockade-linked PD-L1 upregulation on myeloid cells, promoting the expansion of CXCR3 + T cells, and induces gut microbiota compositional changes [53].

In addition to increasing Tregs and promoting IL-10 production in the tumor microenvironment, tumors utilize inhibitory immune signaling pathways through direct cell-to-cell interactions. This involves crucial mediators like CTLA-4 and PD-1, expressed on activated effector T cells, serving as checkpoints to regulate immune activation and proliferation [52]. KD can complement anticancer treatments like immunogenic chemotherapy and immunotherapy, aiming to boost the immune response, minimize the necessity for numerous treatment cycles, and improve the chances of achieving complete remission [53]. In vitro experiments in low-glucose culture media with β-hydroxybutyrate indicated that changes in PD-L1 expression were not due to ketone body supplementation but rather to low glucose levels [54]. Additionally, renal cancer cells exhibit increased PD-L1 expression when glutamine is deprived, mediated through the activation of EGFR/ERK/c-Jun signaling [54]. The effect of a high-fat diet (HFD) on proinflammatory cytokines was investigated in animal models. The study found that an HFD increased cytokine production, including TNF-α, IL-1, and IL-6, in adipocyte cultures but not in peritoneal macrophages. Moreover, HFD-fed animals exhibited decreased mRNA expression of TNF-α and IL-6, along with a reduction in NF-kB expression [55]. However, while some anticancer immunotherapies benefit from very low carb or ketogenic diets, high-fat diets may worsen therapy outcomes [56]. When considering the role of lipids as metabolites, it is essential to recognize their diverse functions in carcinogenesis, encompassing structural roles in membrane formation and energy production. The accumulation of lipid droplets has been positively linked to the advancement of various cancers, including melanoma [57] and pancreatic cancer [58]. Interestingly, in lymphoma, a shift toward lipid utilization is observed, leading to diminished response to treatment [59]. A patient’s nutritional state, especially their fat intake, significantly affects how they respond to anticancer therapy. HFD and HFD-induced obesity shown to accelerate tumor growth [60]. The beneficial anticancer effects of some fats, such as the oleic acid found in olive oil, are also under examination, suggesting that not all fats negatively impact the immune responce against cancer [61].

The gut microbiota’s composition adjusts according to the substrates present in the diet and influences various host functions, including immune modulation. Notably, research has shown that the response to cancer immunotherapy or its treatment-related toxicity can be improved or exacerbated by modulating the gut microbiome [62].

Studies on mouse models have revealed significant shifts in gut microbiome composition following immunotherapy. For instance, mice treated with a single injection of CTLA-4 antibody demonstrated notable alterations, including an increase in Clostridiales and a decline in Bacteroidales and Burkholderiales [62]. Similarly, comparisons of gut microbiome composition in non-small-cell lung cancer (NSCLC) patients receiving Nivolumab with healthy controls revealed marked increases in certain bacterial taxa like Rikenellaceae, Prevotella, Streptococcus, and others in NSCLC patients [63].

The efficacy of CTLA-4 blockade was significantly reduced in mice treated with broad-spectrum antibiotics [62]. In the case of PD-1 blockade, the gut microbiome profiling showed an increase in Bifidobacterium species in mice with delayed tumor growth and effective response to anti-PD-1 therapy [64]. Furthermore, a study comparing the therapeutic effects of ICIs in mice with sarcoma and melanoma treated with antibiotics versus those untreated demonstrated decreased antitumor effects and overall survival in the antibiotic-treated group [65].

Vetizou et al. [62] recolonized antibiotic-treated mice with bacterial species correlated with CTLA-4 Ab-treated intestinal mucosae, which revealed that supplementation with a probiotic containing specific bacterial strains increased the therapeutic response to CTLA-4 Ab.

Kamal & Talib [66] observed that in a mouse model, the application of a combined therapy involving a ketogenic diet (14.1 kcal/2 g) and probiotics (1×10^9 CFU/0.5 ml) significantly reduced tumor size (EMT6/P breast cancer cells implanted in mice) by -55.68% compared to a 106.82% increase in the control group. The potential mechanism of action of probiotics and a ketogenic diet in inhibiting breast cancer in mice involves the modulation of the immune system and the reduction of IGF-1, but requires further investigation. The outcomes of existing studies on the ketogenic diet’s anticancer effects are not unequivocal. In vivo and in vitro investigations have suggested that the observed anticancer effect of the ketogenic diet is attributed to β-hydroxybutyrate. KD prevents and treats colorectal cancer by engaging the hydroxycarboxylic acid receptor 2 with β-hydroxybutyrate, subsequently activating the homeobox only protein signaling pathway to curb colorectal cancer cell proliferation and tumor development [67].

Low-protein diets (LPD), have been implicated in the progression of various types of cancers, demonstrating highly variable effects depending on the cancer type (Fig. 4C). Nevertheless, the precise mechanisms through which dietary restrictions limit cancer progression remain largely unexplored. Research in various mouse cancer models, including lymphoma, melanoma, and colorectal cancer models, indicates that LPD significantly reduces tumor growth, unlike low-carbohydrate diets. This effect depends on the immune system’s functionality, particularly CD8 + T cells and antigen-presenting cells in anticancer immunosurveillance. Depletion of these components or conducting experiments in immunodeficient mice eliminates the LPD beneficial effects. LPD reportedly triggers the unfolded protein response in cancer cells, leading to the activation of the IRE1α and RIG1 signaling pathways [68]. This induces the production of cytokines, stimulating an efficient anticancer immune response. Importantly, this response is not linked to inhibition of cancer cell proliferation or induction of cancer cell death. Rather, it suggests a paradigm shift in understanding the impact of dietary interventions on cancer, highligting the potential of LPDs to modulate cancer cell signaling and the immune response rather than directly suppressing tumors [69].

The link between inflammation, cancer development, and the consumption of specific food groups, particularly dairy products, has sparked scientific debate [70]. Current evidence from the majority of experimental and epidemiological studies suggests the benefits of consuming dairy proteins, without confirmed evidence of carcinogenic effects [71]. Research supports the anticancer effects of selected milk components, including both the casein fraction [70, 71] and whey fraction [72, 73]. For instance, β-lactoglobulin (Lg), the primary component of whey proteins, has shown the stimulation of certain cell proliferation, along with antioxidant [74] and immunostimulatory properties [75]. Lactoferrin (Lf) demonstrates immunomodulatory effects in the innate and adaptive immune response, regulating antibody production and T and B-cell maturation and increasing the percentage of NK cells in the lymphocyte population [76]. Lf has the ability to bind to endotoxins, thereby reducing the production of proinflammatory cytokines [76]. Moreover, Lf enhances the chemotaxis of B, T, and bone marrow-derived dendritic cells toward specific chemokine ligands, indicating its role in immune cell recruitment. Additionally, Lf, along with Lg and their complexes and conjugates, possesses anticancer properties by inhibiting inflammatory cytokine production, promoting immune cell proliferation, and enhancing the NK cell cytotoxicity [77]. The anticancer mechanisms of Lf include cell membrane destruction, induction of apoptosis, cell cycle arrest, and immune response modulation [78]. In a study by Tai et al. [75], administering whey protein isolate (WPI) at 100 mg/kg body weight significantly increased plasma glutathione (GSH) levels and certain cytokines, including IL-2, IL-4, IL-7, and IL-8. These findings suggest that WPI supplementation has immunomodulatory effects, promoting immune cell proliferation and chemotaxis, contributing to enhanced host defense and potential anticancer effects. The antioxidant properties of cysteine and glutamylcysteine groups in WPI may explain its ability to raise GSH levels.

Perrone et al. [79] investigated WPC supplementation’s impact on oral mucositis, the most common side effect of anticancer drugs, in hematopoietic stem cell transplantation (HSCT) patients. Results indicated a significant reduction in mucositis severity and duration for patients consuming WPC at or above 40% of their daily protein requirements. This suggests the potential benefits of WPC in managing side effects in HSCT patients. Studies evaluating the overall effect of dairy intake on fasting blood inflammatory biomarkers found no association between dairy consumption and examined biomarkers, including CRP, IL-1β, IL-6, IL-8, TNF-α, MCP-1, adiponectin, and leukocyte count, even among overweight or obese individuals [80, 81]. Interestingly, there isevidence supporting the anti-inflammatory nature of dairy products or their individual components. In vitro studies have demonstrated that whey protein concentrate (WPC) enhances the antioxidant capacity of cancer cells and induces sensitivity to rapamycin through modulation the cellular redox state and activating GSK3β/mTORC1 signaling in MDA-MB-231 breast cancer cells [81]. Bumrungpert et al. [82] studied the effects of 40 g WPI with selenium and zinc on nutritional status, GSH levels, immunity, and inflammation markers in Thai cancer patients. Results showed improved nutritional status, increased albumin and immunoglobulin G levels, and time-dependent GSH level increases. However, the study lacked a separate control group receiving only WPI, necessitating further investigation to clarify conclusions by examining individual nutritional components separately. Promising results emerged from studies on whey protein hydrolysate (WPH). Experiments with human monocytic leukemia cells (THP-1) revealed potential benefits of WPH on inflammation and endotoxin tolerance by modulating the immune response, and reducing IL-6 and IL-10 levels [83]. An analysis by Kamal et al. [84] showed that camel milk-derived WPH inhibits the production of proinflammatory cytokines like IL-8 and displays antiproliferative activity against liver cancer cells in vitro.

Another layer of complexity stems from the differential effects of LPD on specific amino acids (AAs) in the liver [85]. For instance, LPD is linked to a selective increase in the hepatic levels of aspartate, serine, and glutamate, along with a significant reduction in other AAs. This is intriguing as it challenges the conventional belief that protein restriction uniformly enhances ATF4-activated non-essential amino acid biosynthetic genes [85]. Methionine restriction, a form of LPD, has been studied for its beneficial impact on cancer therapeutics. It leads to an approximate 80% reduction in methionine intake and consistently decreases tumor growth while synergizing with existing cancer therapies. In particular, dietary methionine restriction has been shown to enhance the expression of MHC-I and PD-L1 in cancer cells, indicating an increased capacity for antigen presentation and potential improvement in the immune response to tumors [86].

LPDs have also been linked to the modification of tumor-associated macrophages (TAMs). Dietary protein restriction reportedly alters TAM activity, shifting them toward a more tumoricidal, proinflammatory phenotype (Fig. 5C). This change results in reduced TAM infiltration, decreased tumor growth, and an enhanced response to immunotherapies [87]. Cyclic protein depletion in a *Drosophila melanogaster* intestinal tumor model demonstrated a significant reduction in tumor growth and a return to normal lifespan, suggesting that LPD may offer potential health benefits without associated nutritional drawbacks [88]. In summary, LPD has emerged as a promising dietary intervention in cancer immunotherapy. Beyond its role in restricting tumor growth, LPD appears to modulate immune responses, alter amino acid metabolism, and influence signaling pathways within the tumor environment. This multifaceted impact, demonstrated by several independent research groups, offers potential synergies with existing cancer treatments.

Caloric restriction (CR) involves reducing daily caloric intake by 10–50%, a strategy acknowledged for its effectiveness in extending lifespan and delaying age-related diseases. This phenomenon has been observed across diverse organisms, spanning from yeast to rodents [89–93]. Fasting-mimicking diets (FMDs) seek to replicate fasting benefits with a controlled diet featuring specific macronutrient composition—limited protein, abundant unsaturated fats, and low to moderate carbs. Preliminary results suggest FMDs effectively reduce aging, diabetes, cardiovascular disease, and cancer risk factors without significant adverse effects. Eriau et al. [92] observed that CR, either through fasting or CR mimetics [93], improved the response to anti-PD-1/PD-L1 antibodies when combined with agents inducing immunogenic cell death [94]. This enhancement is attributed to facilitating ATP release from dying cells, stimulating an innate antitumor immune response. Other studies also support CR’s role in reducing the occurrence and severity of specific cancers [95–98]. While promising, the combination of fasting, CR, or their mimetics with immunotherapies remains an area of research that is not been thoroughly explored [99–101]. This integration operates through intricate mechanisms, such as enhancing immunogenic cell death and sensitizing tumor cells (Fig. 5D). The potential benefits support the consideration of incorporating CR and its mimetics into cancer treatment regimens (Table 1).

**Table 1:** Mechanisms and effects of caloric restriction in cancer Immunotherapy.

Mechanism in Caloric Restriction	Effect	References
Autophagy-Dependent Mechanisms	CR stimulates autophagy, promoting ATP secretion and facilitating DC recruitment, a crucial step in mediating an adaptive antitumor immune response. This effect is particularly potent when combined with therapeutic regimens that induce immunogenic cell death.	[89–92]
Inhibition of Tumor Growth	Fasting and CR synergize with tyrosine kinase inhibitors, including crizotinib, in inhibiting tumor growth. Fasting also improves the response to radio- and chemotherapy in mouse glioma models.	[91–93]
Enhanced T-Cell Activity	Energy reduction interventions enhance CD8 + T cell infiltration into the tumor bed while concurrently reducing regulatory CD4 + T lymphocytes.	[93]
Enhancement of Myeloid Cells and DCs:	Energy reduction can enrich mature monocyte-derived DCs, involving critical CD11b+ myeloid cells.	[94]
Sensitization to Immunotherapy	CR, through various mechanisms, can enhance cancer cell sensitivity to T-cell-mediated killing, supporting the idea of combining CR with immunotherapy.	[95, 96]
Combinatorial Strategies	Combining caloric restriction mimetics with ICD-inducing chemotherapy and ICIs improves therapeutic outcomes in preclinical studies.	[94]

### Supplementation of purified compounds and their impact on immunotherapy

In contemporary health practices, it has become evident that attaining therapeutic doses of specific substances solely through regular dietary intake is unrealistic. While a balanced diet is fundamental to a healthy lifestyle, certain nutrients or bioactive compounds crucial for therapeutic benefits often necessitate supplementation. Factors such as food processing, or individual dietary preferences additionally contribute to the challenge of obtaining optimal levels of essential substances solely from food sources [94]. Consequently, the integration of supplementary products - purified natural compounds, has emerged as a pragmatic approach to bridge the gap between dietary intake and body requirements [91]. This acknowledgment highlights the importance of informed supplementation strategies in modern healthcare paradigms.

Vitamin D, a pivotal regulator of immune responses, influences various mechanisms. Polymorphisms in the vitamin D receptor (VDR) gene are linked to autoimmunity and an increased risk of infectious diseases [102], particularly affecting human intestinal T cells and enhancing type 1 immune responses. Vitamin D impacts the differentiation and maintenance of Treg and TH17 cells, crucial for immune response control and implicated in autoimmunity [103]. The generation of Treg cells is promoted by diet-derived SCFAs like butyrate, influenced by the microbiota [104, 105]. Vitamin D also interacts with AhR, involved in mediating IL-22 expression and antimicrobial control in animal models [102]. Overexpressed in various tumors, AhR plays a key role in cancer development and the immune response, making it a promising therapeutic target [106,107]. AhR activation influences the expression of IFN-γ and T-bet, key regulators of type 1 immunity [108]. Vitamin D, further, upregulates MAP kinases, inhibits the NF-kB signaling pathway, regulates cytokine levels, and modulates the interaction between tumor cells and immune cells. It also plays a role in regulating the prostaglandin pathway [109, 110].

On the molecular level, vitamin D binds to IKKβ, a crucial component of the IKK complex, exerting inhibitory effects on the NF-κB signaling pathway [111]. This inhibition modulates cytokine production in various cell types, as evidenced in HEK293 and RAW264.7 cell lines and VDR-deficient mouse embryonic fibroblasts. This interaction prevents the formation of the IKK complex, reducing IKKβ phosphorylation and subsequently inhibiting IKK’s enzymatic activity to phosphorylate IκBα [111]. This leads to reduced IκBα ubiquitylation and degradation. Consequently, the p65/p50 heterodimer is retained in the cytoplasm, resulting in decreased NF-κB transcriptional activity [111]. Zmijewski et al. [112] observed that upon cell stimulation with 1,25(OH)2D3, rapid activation of key signaling pathways, including phospholipase A2 (PLA2) via interaction with PDIA3 and PLAA, as well as the SRC pathway via VDR activation, initiates a cascade involving CAMK2G, PLC, PKC, and culminates in the activation of MAPK1 and MAPK2. Simultaneously, VDR plays a role in downstream regulatory processes triggered by vitamin D, such as the WNT, SHH, and NOTCH signaling pathways, emphasizing the need for more detailed exploration of both genomic and nongenomic effects of 1,25(OH)2D3 and consideration of other potential vitamin D-binding proteins.

The pleiotropic immunomodulatory activity of vitamin D is a subject of in vivo, in vitro, and clinical research. In a retrospective study of 213 melanoma patients who received ICIs, including pembrolizumab, nivolumab, or ipilimumab alone, or a combination of ipilimumab and nivolumab were evaluated. Analyses revealed that pretreatment use of vitamin D, either as therapeutic (ergocalciferol and cholecalciferol) or as part of a multivitamin containing at least 400 IU of vitamin D, significantly reduced the odds of developing ICI-induced colitis. The effect was dose-dependent and most pronounced in patients consuming more than 1000 IU per day [113].

Vitamin D is crucial in modulating the immune response in head and neck squamous cell carcinoma. It impacts the tumor microenvironment, with higher circulating levels linked to increased infiltration of immune cells into tumor tissue, suggesting a potential enhancement of the body’s immune response against cancer cells. Furthermore, vitamin D deficiency is associated with an elevated risk of treatment-associated mucositis and muscle wasting, underscoring its potential therapeutic implications in mitigating treatment-related side effects [114]. In another study, the effect of plasma 25-hydroxyvitamin D (25(OH)D) levels on the survival of metastatic colorectal cancer patients undergoing standard first-line chemotherapy was analyzed. The study found a linear association between 25(OH)D and survival, with a cutoff value of <10 ng/mL identified as the optimal threshold for prognosis. Additionally, the neutrophil-to-lymphocyte ratio (NLR) showed a strong association with vitamin D deficiency and significantly influenced patient outcomes when combined with 25(OH)D levels. These findings highlight the potential prognostic value of vitamin D and its interaction with the immune response [115]. Cusato et al. [109] results appear interesting. As the sole paper on this topic, they presented that during nivolumab therapy for lung cancer, 25(OH)D levels seem to decrease, while 1,25(OH)2D3 levels remain stable. This finding is particularly intriguing due to the interactions of Vitamin D and Nivolumab with PD-1. Vitamin D can directly promote PD-L1 expression, while Nivolumab works by inhibiting PD-1 signaling. This suggests that vitamin D might contribute to the regulation of immune responses by modulating the interaction between PD-1 and PD-L1. However, how Nivolumab may impact 25(OH)D levels remains elusive.

However, it is also important to maintain a stable vitamin D level within the normal level during anti-PD-1 immunotherapy, which improves patients’ prognosis [110].

Vitamin C substantially influences physiological processes in the human body. Its deficiency leads to consequences such as impaired collagen synthesis, reduced iron absorption, and symptoms like easy bruising, purpura, bleeding gums, and muscle pain [116]. Since ascorbic acid is not synthesized endogenously, it must be obtained through food and, if necessary, taken as a dietary supplement.

Studies on ascorbic acid’s anticancer activity have not clearly linked its prophylactic and therapeutic roles in oncology [117]. Two clinical studies, assessing the toxicity of combining bortezomib with arsenic trioxide, ascorbic acid, and high-dose melphalan in multiple myeloma (NCT00661544) and pilot testing intravenous vitamin C in refractory non-Hodgkin’s lymphoma (NHL) (NCT00626444), were discontinued due to poor results. Despite this, vitamin C demonstrated a protective role against chemotherapy toxicity, particularly in stage III and IV ovarian cancer, showing decreased 1st- and 2nd-degree toxicity in patients treated with carboplatin and paclitaxel along with intravenously administered vitamin C [118]. It is challenging to definitively attribute this effect to vitamin C alone. Antioxidant compounds, such as N-acetylcysteine in the rat model of pediatric tumors [119], or vitamin E countering chemotherapy-induced peripheral neuropathy [120], may similarly reduce chemotherapy side effects. Clinical trials focusing on vitamin C in immunology are limited, with no published results or observations, as seen in trials like Impact of HLNatural Immune Supplement on Colds (NCT04103099) or Nutrition Interventions to Support the Immune System in Response to Stress (NCT02053506). Research suggests that single doses of high-dose vitamin C (15–25 g) can enhance blood antioxidant capacity without causing oxidative damage to plasma proteins and lipids. However, doses exceeding 25 g have the opposite effect, reducing total antioxidant capacity [121]. The renewed interest in vitamin C as a potential cancer treatment has led to studies demonstrating improved patient quality of life and potential synergistic effects with other treatments. Despite positive findings, challenges such as the lack of patentability, historical controversies, and unclear mechanisms of action impede large-scale trials [122, 123]. Importantly, vitamin C is not recommended as a standalone monotherapy but rather as a supportive compound, potentially alleviating inflammation in patients with antioxidant deficiency symptoms during conventional oncological therapies.

Most conclusions and hypotheses are based on in vitro studies and animal models, which do not fully reflect human body complexities [124]. In cellular studies, vitamin C significantly influences lymphocytes, especially T cells and NK cells, with unclear effects on B lymphocytes. Vitamin C supports NK cell proliferation, but its impact on NK cell function remains uncertain [125, 126]. Experimental data suggests it may enhance immune checkpoint therapies (ICTs) in cancer treatment, modulating gene expression and immune cell differentiation. Its anticancer effects, particularly at high intravenous doses, include pro-oxidant effects and disrupting iron metabolism in cancer cells. This implies vitamin C could be a safe and effective addition to ICT, potentially expanding benefits to more patients [125] and playing a significant role in immunomodulation (Table 2).

**Table 2:** Recent in vitro research on vitamin C’s roles in immune regulation and cancer treatment revelations.

Key findings	Mechanism	References
Enhances functionality of immune cells	NK cells, and Vγ9Vδ2 γδ T cells, suggesting potential in adoptive immunotherapy	[117–120]
Stimulates DCs to secrete more IL-12	Driving naïve CD4 + T cells to differentiate into Th1 cells and boosting CD8+ memory T-cell production in vivo	[121, 122]
Regulates plasma cytokine levels and anti-inflammatory mechanisms	Hindering tumor development through cytokine release modulation and NF-κB activation	[121–123]
Exhibits synergistic effect with anti-PD-1 therapy	Enhancing immune cell infiltration and cytotoxic activity in the tumor microenvironment, it also collaborates with anti-PD-1 and anti-CTLA-4 treatments, potentially boosting antitumor immunity.	[124]

These effects involve specific signal transduction pathways associated with vitamin C. However, comparing these results with clinical data is challenging; to date, such comparisons are quite limited and, for safety reasons, often unfeasible to implement [127–134].

**Fig. 6:**
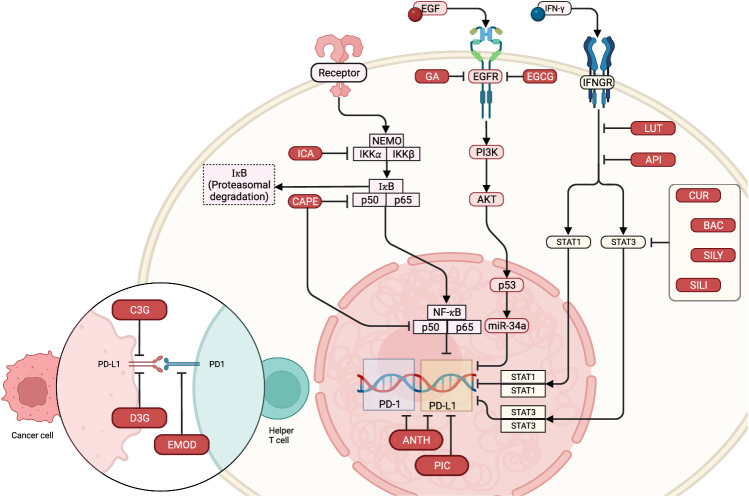
Modulation of PD-L1 and PD-1 expression by phytochemical and phenolic compounds. Each compound, including ANTH (anthocyanin), API (apigenin), BAC (baicalein), CAPE (caffeic acid phenethyl ester), C3G (cyanidin-3-O-glucoside), CUR (curcumin), D3G (delphinidin-3-O-glucoside), EGCG (epigallocatechin gallate), EMOD (emodin), GA (gallic acid), ICA (icaritin), LUT (luteolin), PIC (piceatannol), SILI (silibinin), and SILY (silymarin), inhibits specific signaling pathways resulting in the downregulation of PD-L1 or PD-1 expression in cancer cells. Created with BioRender.com.

Polyphenolic compounds such as apigenin, luteolin, anthocyanin, cyanidin-3-O-glucoside, silymarin (silibinin), epigallocatechin gallate (EGCG), hesperidin, icaritin, and baicalein (baicalin) exhibit diverse biological effects, including antioxidant, antimicrobial, anticancer, and anti-inflammatory activities. They are extensively researched for their capacity to regulate PD-L1 expression, a surface protein pivotal in suppressing immune responses against cancer cells (Fig. 6). Combined and reduced wordiness: Apigenin and its metabolite luteolin inhibit IFN-γ-induced PD-L1 expression in human and mouse breast cancer cells by suppressing STAT1 activation [135]. This effect extends to melanoma, as apigenin and curcumin treatment significantly suppresses tumor growth by inhibiting PD-L1 expression in melanoma cells [136, 137]. Anthocyanin and its metabolites significantly inhibit both PD-1 and PD-L1 expression, promoting an immune response and suppressing colon cancer progression [138, 139]. This effect is associated with modifications in the gut microbiome, enhancing the production of anticancer and anti-inflammatory SCFAs [138]. Additionally, cyanidin-3-O-glucoside (C3G) inhibits PD-L1 in colon cancer cell lines [139]. Silymarin and its primary bioactive flavonolignan, silibinin, inhibit PD-L1 in cancer cells by suppressing STAT3 signaling [140]. EGCG, a potent antioxidant in green tea, suppresses PD-L1 expression in NSCLC cells by inhibiting the JAK2/STAT1 and EGF receptor (EGFR)/Akt signaling pathways [141]. Hesperidin and icaritin exhibit potential in suppressing PD-L1 expression in various cancer cells, including estrogen-dependent triple-negative breast cancer and androgen-dependent prostate cancer cells. They achieve this by inhibiting key signaling pathways, downregulating Akt and NF-κB, and hindering cell migration through MMP-9 and MMP-2 inhibition, suggesting roles in ICI therapy [142–144]. Baicalein and its conjugate, baicalin, regulate PD-L1 expression in liver cancer cells, leading to increased T-cell-mediated cancer cell death [144]. These phytochemicals may offer therapeutic potential in enhancing ICI therapy effectiveness. Notably, the modulation of PD-L1 expression extends beyond flavonoids to other phenolic compounds. Curcumin, from turmeric, inhibits NF-κB and STAT3 signaling, attenuating tumor progression and enhancing anticancer immunity by stabilizing PD-L1 and reducing CSN5 activity, a deubiquitinase modulating PD-L1 ubiquitination [145, 146].

Gallic acid (GA), a phenolic acid, inhibits the PI3K/AKT pathway, upregulating p53 and miR-34a, which suppresses PD-L1 expression [147]. Emodin, an anthraquinone, with anti-inflammatory properties, attenuates PD-L1 stabilization, decreasing PD-1 binding and enhancing T-cell-mediated tumor cell death [148]. Caffeic acid phenethyl ester (CAPE), from honeybee products, exhibits diverse antitumor mechanisms. In ovarian cancer cell lines, CAPE decreases SKOV-3 cell viability and invasiveness, promoting apoptosis. In vivo, it inhibits ovarian cancer growth, suppressing proliferation markers Ki67 and PCNA. CAPE’s anticancer activity is linked to NFκB pathway suppression, inhibiting IκB phosphorylation, p65 nuclear translocation, and NFκB p65 DNA binding activity [149]. In inflammation-stimulated breast cancer cells, CAPE and ethanol-extracted Chinese propolis (EECP) inhibit proliferation, induce apoptosis, activate autophagy, and downregulate the TLR4 signaling pathway [150]. CAPE and EECP may have therapeutic potential in treating inflammation-induced tumors [151, 152]. CAPE also suppresses PD-L1 expression induced by Epstein‒Barr virus latent membrane protein 1 (LMP1) in nasopharyngeal carcinoma (NPC), suggesting its potential use to block the LMP1 oncogenic pathway and PD-1/PD-L1 checkpoints in EBV-positive NPC patients [152]. Polyphenolic compounds primarily modulate PD-L1 expression by inhibiting key signaling pathways (IFN-γ-induced STAT1, STAT3, JAK2/STAT1, EGFR/Akt, and NF-κB), potentially enhancing the immune system’s capacity to target and eradicate cancer cells [153].

Polyphenols also significantly enhance monoclonal antibody therapies like trastuzumab [154] and cetuximab [152]. For instance, anthocyanins cyanidin-3-glucoside and peonidin-3-glucoside inhibit trastuzumab-resistant breast cancer cells in vitro and in a murine xenograft model in vivo [154]. The codelivery of paclitaxel and 5-demethylnobiletin, a hydroxylated polymethoxyflavone from citrus, in cetuximab-functionalized nanostructured lipid carriers effectively inhibits tumor growth in a model of paclitaxel-resistant cell-bearing mice [152]. Our previous review offers further insights into the role of polyphenols in cancer therapy [155].

*Perilla frutescens* (L.) is a versatile plant in the Lamiaceae family, commonly used in East Asian countries as a traditional herbal medicine and, utilized to treat various illnesses such as cough, respiratory tract infections, food poisoning or diarrhea. It exhibits a diverse array of chemicals, including volatile oils like limonene, linalool, farnesene, and germacrane, contributing to its distinct aroma. The flavonoid content is characterized by compounds like luteolin, apigenin, and phenolic acids such as caffeic acid and rosmarinic acid. Compounds such as perilla ketone, isoegomaketone, and perillaldehyde are currently being studied for potential anticancer and anti-inflammatory properties [156]. However, the biological effects of *P. frutescens* on human neutrophils and the molecular mechanisms underlying these effects remain poorly understood [157]. Perilla seed oil is regarded as one of the premier plant sources of omega fatty acids, comprising approximately 53–62% α-linolenic acid (ALA, 18:3, ω-3), 10–13% linoleic acid (LA, 18:2, ω-6), and 11–16% oleic acid (18:1, ω-9), with a mean ω-6/ω-3 ratio of about 0.20 [158].

Chen et al. explored the inhibitory effect of Perilla frutescens on N-formyl-Met-Leu-Phe (fMLF)-stimulated human neutrophils. Observations indicated that the extract, at concentrations of 1, 3, and 10 µg/ml, significantly reduced superoxide anion production, elastase release, reactive oxygen species formation, CD11b expression, and cell migration in a dose-dependent manner. The extract suppressed the fMLF-induced phosphorylation and enzymatic activity of Src family kinases, including Src and Lyn, and attenuated intracellular Ca2+ levels, thereby inhibiting the inflammatory activation of neutrophils [157].

Controlled apoptosis induction in genetically modified T-cells through the use of small molecule drugs. Selectively eliminating the modified T-cells after they have exerted their therapeutic effect reduces their potential for toxicity (inflammatory factor storms and unpredictable off-target and organ-specific toxicity). The enzyme CYP4B1, a mammalian cytochrome P450 monooxygenase, plays a crucial role in this process [159]. Roellecke et al. was discovered that perilla ketone is more potent than 4-ipomeanol in inducing apoptosis in genetically modified T-cells expressing the CYP4B1P + 12 suicide gene. The key difference is the presence of a methyl group at C5 in perilla ketone, as opposed to a hydroxyl group at the same position in 4-ipomeanol. This structural variation likely underlies the differential interaction with the CYP4B1P + 12 enzyme, contributing to perilla ketone’s efficacy in triggering apoptosis [160].

Linette et al. demonstrated the apoptosis-inducing activity of perilla ketone in ACT. Concentration of only 2.9 mmol L^−1^ perilla ketone resulted in the death of 84 ± 1.08% of ΔNGFR-T2A-CYP4B1P + 12-transduced T-cells in vitro, with minimal toxicity to primary non-transduced T-cells. Remarkably, perilla ketone presented 3–5 times higher activity than 4-ipomeanol, inducing apoptosis in transduced cells at a faster rate and at lower concentrations, suggesting its potential to mitigate the toxicity associated with ACT [161]. Thesseling et al. explored how the CYP4B1 enzyme activates perilla ketone and 4-ipomeanol, revealing that the furan functional group of 4-ipomeanol is activated by epoxidation. In contrast, perilla ketone can undergo two chemical reactions under CYP4B1: epoxidation of the furan functional group or hydroxylation of the isopropyl functional group. Either of these reactions promote the activation of perilla ketone, suggesting its more versatile mechanism of action [162].

### Beverages compounds impact on immunotherapies

Polyphenolic compounds found in various beverages, including tea, coffee, and red wine, have garnered significant attention for their potential health benefits, including their impact on cancer immunotherapy. Green tea contains antioxidants and catechins, which have been shown to have anti-inflammatory and potential anticancer effects - EGCG, as mentioned earlier, and caffeine - present also in coffee. Mentioned earlier, polyphenolic compound, found in red wine, has gained attention for its potential impact on cancer treatment and immunotherapy approaches–resveratrol [163].

EGCG has demonstrated its ability to inhibit tumor growth and proliferation by downregulating MAPK, ERK, and Akt activation inducing cell cycle arrest [164]. In addition to its previously mentioned effect on PD-1L, there is also evidence indicating that combined treatment with immunomodulatory doses of EGCG can boost the immune response of specific CD8 + T lymphocytes and CD4 + Th1 lymphocytes induced by DNA vaccine, thereby offering long-term protection against cancer [165]. Moreover, EGCG seems to not only increase the proportion of CD8 + T lymphocytes in vitro but also significantly enhances the CD4 surface intensity on activated T lymphocytes and promotes the Th1 response when combined with resveratrol [156]. Microparticle vaccines employing EGCG and aluminum ions as ligands to encase individual tumor cells efficiently stimulated dendritic cells (DCs) and notably augmented the production of Th1-related cytokines, leading to immunotherapeutic effects [156]. There is already review focusing on immunomodulatory potential of EGCG [164].

Caffeine, present in coffee and tea, is a general adenosine receptor antagonist which at physiologically relevant concentrations preferentially antagonizes the A2AAR. Jin et al. [166] explored the immunomodulatory effects of CD73, which increased expression on tumors negatively influences tumor antigen-specific T cell immunity. There has been considerable interest in understanding the role of CD73-mediated generation of extracellular adenosine in host defense mechanisms, given adenosine’s well-known anti-inflammatory properties. Notably, studies have shed light on the A2A adenosine receptor (A2AAR)-mediated ‘adenosinergic pathway’ as a critical regulator of immune responses in vivo [167]. Interestingly, the survival of mice treated with T cells and caffeine exceeded that of mice treated with caffeine alone or T cells alone. These findings support the notion that blocking the tumor CD73-adenosine-A2AAR pathway restores tumor-specific immunity and enhances the efficacy of adoptive T-cell therapy [166]. Additionally, Tej et al. [168] investigated the effect of caffeine and anti-PD1 mAb combination therapy, which showed enhanced antitumor activity than caffeine or anti-PD1 mAb monotherapy. The blockade of adenosine-A2Areceptor pathway by caffeine and blockade of PD1 pathway by anti-PD1 mAb increased the infiltration of total T lymphocyte population, possibly due to combined blockade of both the pathways.

An interesting compound to investigate in immunotherapy approaches could be quinine present in gin and tonic, Quinine was demonstrated to suppress the expression of BCL‑2, whilst stimulating that of BAX in a time‑dependent manner. The mutual antagonism that exists between subsets of BCL‑2 family members, including BCL‑2 and BAX, directly impacts the mitochondrial membrane permeability, which ultimately determines whether cytochrome c is released in order to activate caspases and induce apoptosis [169]. Liu et al. [170] study identified quinine as a ligand which can bind effectively with TRAF6, and subsequently inhibit AKT activity and phosphorylation, thereby promoting quinine‑induced apoptosis.

Resveratrol (RSV) and its derivative piceatannol can manipulate PD-L1 expression in cancer cells. RSV disrupts N-linked glycosylation, leading to abnormal PD-L1 glycosylation and enhanced T-cell activity against cancer cells. These compounds modulate PD-L1 expression, with RSV significantly increasing PD-L1 levels in Cal51 breast cancer cells and piceatannol upregulating PD-L1 in HCT116 colon cancer cells. Used together, they synergistically amplify PD-L1 expression, especially in cancer cell lines with low PD-L1 expression. This novel therapeutic strategy may enhance the effectiveness of PD-1/PD-L1 axis-targeting therapies. PD-L1 induction is regulated transcriptionally via the NFκB pathway, evidenced by p65 subunit nuclear accumulation upon RSV treatment. The IKK inhibitor BMS-345541 and histone modification inhibitors like resminostat inhibit PD-L1 induction. These treatments negatively impact tumor cell survival, as indicated by γH2AX upregulation, caspase 3 cleavage, survival marker downregulation, and G1-to-S cell cycle arrest [153].

Polydatin, a resveratrol precursor, promotes apoptosis and inhibits cancer cell proliferation by upregulating miR-382 and suppressing PD-L1 expression [171, 172]. However, miRNA’s role in cancer is complex, varying with cellular context, as miRNAs regulate diverse target genes and cellular pathways [173].

### High-salt diet impact on immunotherapy

Recent studies on dietary salt’s impact on macrophage function within tumor microenvironments highlight its significant influence on macrophage plasticity, revealing the intricate relationship between high salt conditions and immune modulation in cancer progression [173, 174]. In mouse models, researchers found that a high-salt diet (HSD) increases local sodium chloride concentrations in tumor tissues, inducing substantial osmotic stress, which suppresses the production and accumulation of myeloid-derived suppressor cells (MDSCs) in blood, spleen, and tumors. Consequently, monocytic MDSCs transform into anti-tumor macrophages, while granulocytic MDSCs adopt pro-inflammatory roles, reinvigorating T cell anti-tumor activities. Moreover, HSD enhances the expression of p38 mitogen-activated protein kinase-dependent nuclear factor of activated T cells 5 during M-MDSC differentiation [175]. Elevated sodium chloride levels also increase the expression of epithelial sodium channels in breast cancer cell lines (MDA-MB-231 and MCF7), guiding toward a less aggressive phenotype.

Amara et al. [175] observed that IL-17, combined with high sodium chloride levels, synergistically induced reactive nitric oxide species in breast cancer cell lines. This induction, facilitated by the upregulation of inducible nitric oxide synthase, signifies an inflammatory response potentially aiding cellular adaptation against apoptotic signals. It is associated with increased expression of anti-apoptotic genes like BCL2 and BCL-xL, suggesting a protective mechanism mediated by NF-κB signaling pathways [175]. Cancer cells adapt to high salt conditions by intensifying the Warburg effect, by heightened glucose consumption and lactate production, indicating a shift towards glycolytic activity. This metabolic adjustment suggests that elevated sodium levels directly support a metabolic phenotype favoring cancer cell growth and survival in hypoxic environments [175]. Moreover, high sodium concentrations profoundly influence the immune landscape of the tumor microenvironment, and shifts towards an anti-tumor response. This is characterized by reduced levels of immunosuppressive cytokines such as IL-10 and TGF-β, and increased pro-inflammatory cytokines like IL-12 and IFN-γ, which enhance the recruitment and effector functions of cytotoxic lymphocytes and antigen-presenting cells [176].

High salt concentrations also impact tumor stroma and vascular networks, suggesting an indirect influence on angiogenesis and tumor invasion [177]. Studies suggests that high salt conditions in culture media prompt the specific differentiation of peripheral blood mononuclear cells toward an M2 macrophage phenotype (CD11b+CD14lowCD16 + ), indicating that high salt environments, similar to those observed in tumor microenvironments, drive the accumulation of anti-inflammatory M2 macrophages, negatively correlated with cancer prognosis [173, 175]. This differentiation is influenced by the cytokine milieu within the tumor environment; IFNγ and TNFα promote an M1 phenotype, while TGFβ encourages the M2 phenotype. Furthermore, Jantsch et al. [174] demonstrated that high salt conditions could trigger an M1 phenotype switch in macrophages, offering protection against infections like Leishmania, suggesting the macrophage response to salt is context-dependent and varies with the microenvironment.

Overconsumption of salt may impact the immune system by altering the balance of immune cells towards a state that promotes inflammation. Nevertheless, it’s essential to approach this with caution, considering the adverse effects of a high-salt intake on organism, which can lead to high blood pressure, heart disease, and stroke.

### Conclusions and future prospects

A crucial question arises: to what extent can boosting the immune system advance anticancer therapy? Considering demographic trends, environmental factors, and changing dietary habits, can we realistically anticipate a significant reduction in cancer incidence? Anticipated progress in immunotherapy holds the potential to transform the oncology landscape.

Leveraging genomic technologies, such as next-generation sequencing (NGS), along with other omics techniques like proteomics and metabolomics [178–180], will enable more precise tumor profiling and pave the way for personalized immunotherapies. Growing interest in machine learning models for biomarker research [181] adds another layer of potential.

The escalating global cancer burden, influenced by factors like extended life expectancy, environmental pollution, and genetically modified food, demands ongoing research for more targeted treatments. Immunotherapy, in certain instances, stands as the first-line immuno-oncology monotherapy, with chemotherapy/radiotherapy scheduled for future use. The distinct mechanism of action compared to cytostatics or radiation allows potential exploitation in adjunct therapy or to alleviate side effects. Looking ahead, a customized combination of these therapies, attuned to individual patients and specific cancer characteristics, may optimize treatment outcomes and address the burgeoning cancer prevalence across age groups.

These therapies, marking a revolutionary impact on cancer treatment, exhibit promise across various malignancies, enhancing survival rates. Ongoing research aims to refine these immunotherapies and formulate strategies for their integration with other treatments, ushering in a more personalized approach to cancer treatment that considers patient genetic and immunological profiles. Simultaneously, the role of lifestyle factors, such as diet and exercise, in modulating the immune system gains prominence, underscoring the importance of a holistic approach to cancer care encompassing both prevention and treatment.

Furthermore, integrating immunotherapies with conventional treatments has the potential to synergistically enhance overall cancer therapy efficacy. These advances provide a glimpse into a future where immunotherapy assumes an increasingly central role in cancer care. As clinical research progresses, there is anticipation that immunotherapy will become an integral part of the standard approach to cancer treatment. Depending on the cancer type and stage, immunotherapy might serve as a first-line treatment, complement primary therapy, or address cases of treatment resistance. Due to its generally lower toxicity compared to chemotherapy or radiotherapy, the use of immunotherapy is likely to rise.

A well-suited dietary regimen can significantly boost the immune system for both cancer prevention and as a complementary approach to therapy. Ongoing research actively explores specific diets and supplements with the potential to enhance immunity and complement anticancer treatments. This dual-focused approach, marrying cutting-edge therapies and lifestyle considerations, underscores the multifaceted strategy required for effective cancer care in the evolving landscape of medical research and treatment.

There is a limited portfolio of ongoing clinical trials (clinicaltrials.gov database) investigating polyphenol-assisted immunotherapy, with approved trials yet to publish their findings. Notable trials include investigations into the effect of purified natural compounds on various cancers (NCT03994055, NCT03959618, NCT03824652), the utilization of chlorogenic acid for advanced lung cancer and glioblastoma (NCT03751592, NCT02728349, NCT02245204), and the exploration of the effectiveness of combining whole brain radiotherapy with silibinin in managing brain metastases (NCT05793489). Another study examined a new immunomodulatory agent, icaritin, an active ingredient of the Chinese herb Epimedium, combined with other therapies for hepatocellular carcinoma (NCT05903456). Although challenging to extrapolate preclinical data to clinical applications, the rising interest in these trials highlights the potential of polyphenols in augmenting cancer immunotherapy.

The emergence of cancer immunotherapy signals a future direction in cancer treatment, emphasizing curative therapies over extending patients’ lifespan. The primary focus of cancer immunotherapy has been on effectively inducing and augmenting the immune response against cancer. However, the intricate nature of the cancer immunity cycle poses theoretical challenges for success through this approach alone. The usefulness of ICIs, the mainstay of cancer immunotherapy, has only partially covered the cancer immunity cycle. In the future, addressing challenges in each phase of the cancer immunity cycle and exploring combination therapies are crucial to ensuring efficient functioning.

The mechanism of action of cancer immunotherapy differs significantly from conventional cancer drugs, requiring treatment methods based on a thorough understanding of its characteristics. The future development of combination therapy involving ICIs holds promise, potentially achieving not only additive but also synergistic effects. The history of conquering cancer by cancer immunotherapy has begun, necessitating long-term and persistent efforts to achieve a cure for cancer. Continuing basic research and clinical development is crucial.
